# New Insights Into Diuretic Use to Treat Congestion in the ICU: Beyond Furosemide

**DOI:** 10.3389/fneph.2022.879766

**Published:** 2022-07-08

**Authors:** Victor Joaquin Escudero, Jordi Mercadal, Alícia Molina-Andújar, Gaston J. Piñeiro, David Cucchiari, Adriana Jacas, Albert Carramiñana, Esteban Poch

**Affiliations:** ^1^ Nephrology and Kidney Transplantation Department, Hospital Clínic, Institut d'Investigacions Biomèdiques August Pi i Sunyer (IDIBAPS), University of Barcelona, Barcelona, Spain; ^2^ Surgical Intensive Care Unit, Anesthesiology Department, Hospital Clínic, Institut d'Investigacions Biomèdiques August Pi i Sunyer (IDIBAPS), Univesitat de Barcelona, Barcelona, Spain

**Keywords:** diuretics, fluid overload, congestion, intensive care unit, acute kidney injury

## Abstract

Diuretics are commonly used in critically ill patients with acute kidney injury (AKI) and fluid overload in intensive care units (ICU), furosemide being the diuretic of choice in more than 90% of the cases. Current evidence shows that other diuretics with distinct mechanisms of action could be used with good results in patients with selected profiles. From acetazolamide to tolvaptan, we will discuss recent studies and highlight how specific diuretic mechanisms could help to manage different ICU problems, such as loop diuretic resistance, hypernatremia, hyponatremia, or metabolic alkalosis. The current review tries to shed some light on the potential use of non-loop diuretics based on patient profile and give recommendations for loop diuretic treatment performance focused on what the intensivist and critical care nephrologist need to know based on the current evidence.

## Introduction

Diuretic use in the intensive care unit (ICU) has been controversial due to the fear of inducing or aggravating hypovolemia, especially in hemodynamically unstable patients under vasopressor use or with concomitant acute kidney injury (AKI). This long-lasting belief stems from the conception that hypovolemia has a central role in the development of AKI during ICU stay as a causative factor or as an important contributor to septic AKI, the most frequent form of AKI in the ICU (1).

Publications during the last 20 years have shifted the focus away from hypovolemia as the main or more important causative agent of AKI, and we now know that AKI is a complex syndrome with many different causes and complex pathophysiological pathways, not entirely understood, that needs a more personalized and focused approach (2). It has been a common practice in the ICU to administer fluids as a reflex to undifferentiated hypotension, oliguria, rising creatinine, or high lactate (3). That practice was reinforced with the results of the River’s study of early goal-directed therapy in septic patients in 2001 (4) that heavily influenced the first Surviving Sepsis Campaign Guidelines in 2004 and, since then, all the hemodynamically management in the ICU (5). Early goal-directed therapy has finally been shown as a flawed concept after three big randomized controlled trials (RCTs) had shown no benefit with respect to conventional resuscitation in the ICU (6). However, a liberal fluid approach is still widely used and advocated on the basis of the deep influence that early goal-directed therapy has had during the last 20 years.

Liberal use of fluids is accompanied by the hard price of congestion. Lung congestion has been a feared enemy in the ICU and a landmark study in 2006 showed for the first time that a conservative approach to fluid management in patients with mild acute respiratory distress syndrome was beneficial in terms of ventilator-free days with no more AKI and a tendency to reduce the use of renal replacement therapy (7). Since then a number of observational data have consistently shown that a positive fluid balance is an independent risk factor of mortality in ICU patients and especially the ones with AKI with high central venous pressure (8), as a surrogate of congestion, playing a potential role in the development or worsening of AKI (9, 10). That link has grown lately with the advent of point of care ultrasound to evaluate not only lung but also systemic congestion through assessing venous Doppler tracings of the inferior vena cava, suprahepatic, portal, and renal veins with the recently described venous excess score that has shown association between systemic congestion and AKI development in post cardiac surgery patients and cardiorenal syndrome (11, 12).

Congestion is an everyday concern in the ICU, and despite the fact that most data are of observational nature, diuretic use seems to be beneficial if it avoids/limits congestion with the desired fluid balance in patients with AKI (13, 14).

Many questions remain open and good evidence from prospective RCTs await to define the role of diuretics in the ICU, but we feel that having a more profound pharmacological knowledge of the different types of diuretic drugs and their use in typical clinical scenarios in the ICU can be of great value to the critical care/nephrology clinicians.

## Carbonic Anhydrase Inhibitors

### Mechanism of Action

Carbonic anhydrase (CA) is an enzyme found in red blood cells and other tissues including the kidney. Fourteen isoforms have been identified, two of which are fundamental in acid–base homeostasis by the kidney: type II, which is found in the cytoplasm of epithelial cells of the proximal tubule and in the intercalated cells of the distal part of the nephron (known as “aldosterone-sensitive distal nephron” [ASDN]); and type IV, which is found in the luminal and basolateral portion of the cell membranes of proximal cells, as well as in the thick portion of the ascending limb of the loop of Henle and in intercalated “alpha” cells of ASDN (15, 16). Its main action is in the proximal tubule of the kidney, where it allows the reabsorption of bicarbonate, sodium, and chloride.

**Figure 1:**
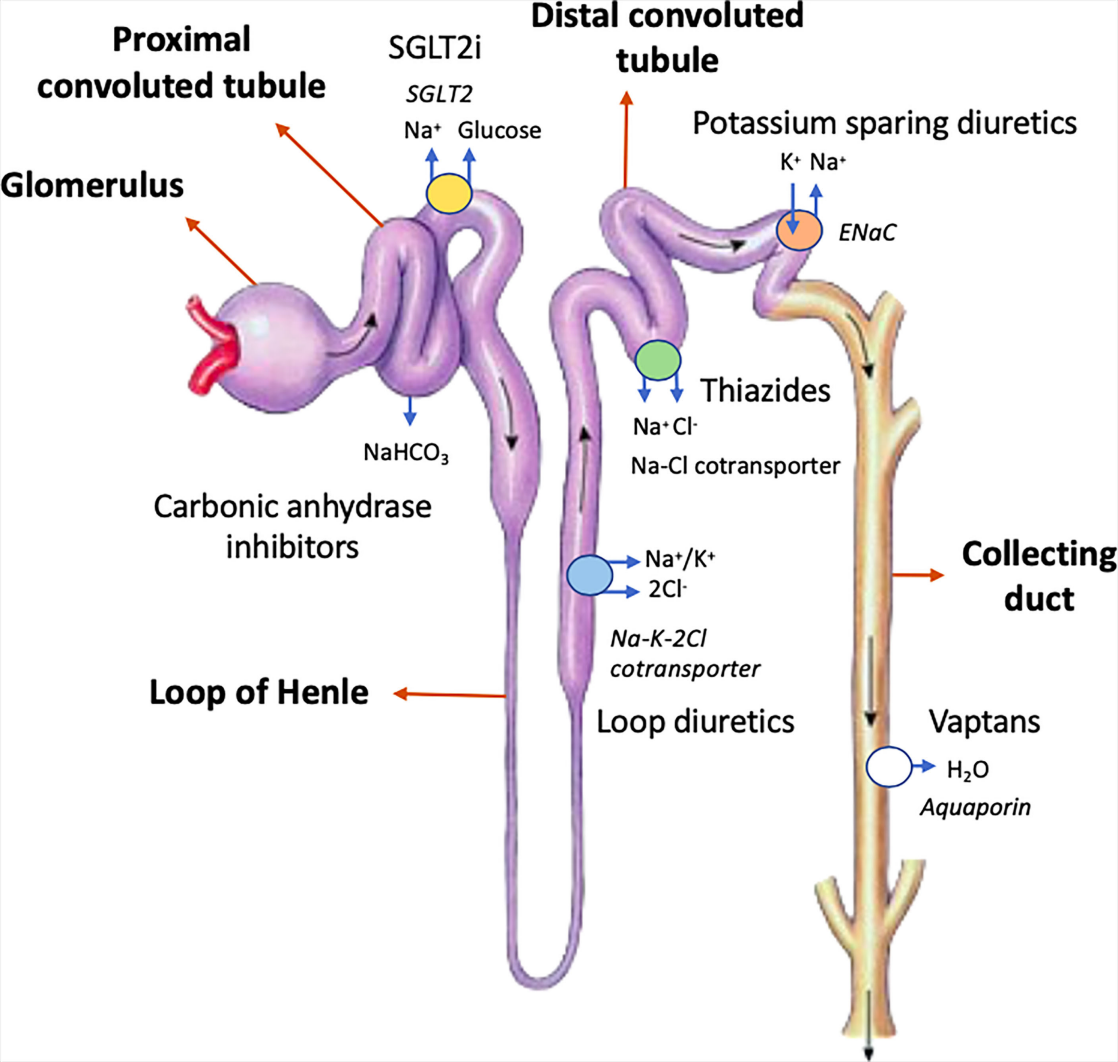
Sites of diuretic action in the nephron. “K^+^-sparing agents” refers to the epithelial sodium-channel inhibitors (e.g., amiloride and triamterene) and mineralocorticoid-receptor antagonists (e.g., spironolactone and eplerenone). NaHCO_3_ denotes sodium bicarbonate. Na^+^ refers to sodium ions, K^+^ refers to potassium ions, and Cl^-^ refers to chlorine ions. H_2_O refers to water. ENaC refers to epithelial sodium channel.

Carbonic anhydrase inhibitors (CAI) block the action of this enzyme. The main example of this type of diuretic is acetazolamide. By inhibiting this enzyme, the main effect is that sodium bicarbonate is excreted along with excess water. In response, a reabsorption of ammonia (NH3^+^) occurs at a more distal level, ultimately resulting in metabolic acidosis. The diuretic effect is weak, but it can enhance the loop diuretics’ effect when used together since more sodium arrives into the thick ascending limb of the loop of Henle. Additionally, acetazolamide has intrinsic renal vasodilatory effects, protecting the nephron against ischemia–reperfusion damage. Finally, it also blocks bicarbonate reabsorption along the distal tubule by acting on ASDN (17, 18). The reader can find the sites of diuretic action in the nephron in **Figure 1**.

It is known that the use of this diuretic generates tolerance when used for more than 48 h, with a decrease in natriuresis; thus, it is not recommended to use it for more than 3–4 days per week or for more than two consecutive days in order to sustain its desired diuretic properties (19).

The pharmacokinetic characteristics and dosage of acetazolamide are shown in **Table 1** (20–22).

**Table 1:** Pharmacokinetic characteristics and dosage of acetazolamide.

Drug	Bioavailability	Half-life	Duration of effect	Dosage
Acetazolamide	60%–100% orally	6–10 h	8–12 h	250–500 mg qd for 48 h

Qd, once a day.

### Potential ICU Indications

In case of fluid overload, acetazolamide used in combination with loop diuretics is an option to explore. The DIURESIS-CHF study included 34 acute heart failure patients on loop diuretics with fluid overload. All of them had a serum sodium concentration <135 mEq/L and/or serum urea/creatinine ratio > 50 and/or an admission serum creatinine increase of >0.3 mg/dl compared to baseline. Patients were randomized to receive acetazolamide 250–500 mg daily plus bumetanide or high-dose bumetanide. The primary end point was natriuresis after 24 h. The study found that addition of acetazolamide increased the natriuretic response to the loop diuretic compared to an increase in loop diuretic dose, but there was a non-significant trend towards lower all-cause mortality or readmissions for heart failure (23). On the contrary, acetazolamide use was associated with a trend towards higher decline in renal function during decongestive treatment (>0.3 mg/dl rise in creatinine within 72 h). Urinary output was not described and the study did not specify if patients were admitted in critical care units.

In the ICU setting, metabolic alkalosis is a common finding, and it is associated with worse outcomes (24). It is frequently iatrogenic, owing to the administration of diuretics or steroids but also as a result of permissive hypercapnia by lung protective strategies, chronic obstructive pulmonary disease (COPD) patients, or nasogastric suctioning. Therefore, apart from its potential use in diuretic resistance, acetazolamide could be useful when metabolic alkalosis coexists, especially that caused by loop diuretics. Also, theoretically, by correcting metabolic alkalosis, it may increase minute ventilation and improve oxygenation, which could facilitate weaning from mechanical ventilation (25). However, in 2016, Faisy et al. published the first RCT evaluating the effect of acetazolamide vs. placebo on the duration of invasive mechanical ventilation in patients with COPD, and found no differences (26).

We can conclude that in the ICU setting, acetazolamide could play a role in the treatment of congestion, especially when high doses of loop diuretics are used and associated with metabolic alkalosis. In other context, such as COPD with mixed pH disorders, it could be considered when congestion is present.

### Adverse Events

Acetazolamide may worsen hyponatremia, hypokalemia, and metabolic acidosis due to its mechanism of action. Patients who take acetazolamide on a long-term basis are at increased risk of developing calcium phosphate lithiasis due to increased urinary pH and decrease in urinary citrate (27). That should not be a problem in the ICU setting unless patients already have stones.

Likewise, other effects that may be caused are those related to the sulfonamide family, such as immunological reactions with various skin manifestations (toxic epidermal necrolysis Stevens–Johnson syndrome), hepatic toxicity (fulminant hepatitis, increased risk of encephalopathy), anaphylaxis, or blood dyscrasias (agranulocytosis, thrombocytopenia, aplastic anemia, and thrombocytopenic purpura).

For all these reasons, its use is not recommended in patients with advanced liver disease, or in patients with hyponatremia, hypokalaemia, and metabolic acidosis (27).

## Osmotic Diuretics

### Mechanisms of Action

Osmotic diuretics are freely filtered at the glomerulus but poorly reabsorbed in the tubules. Mannitol is the main example of this group, and its mechanism of action is completely different from the other diuretic agents as they do not directly interfere with electrolyte reabsorption mechanism. In the proximal tubule and loop of Henle, mannitol increases osmolarity and, consequently, tubular fluid reabsorption is diminished. Furthermore, other mechanisms play a role in its diuretic action. Its hemodilution effect increases total renal blood flow, and at the same time, the decrease in plasma colloid osmotic pressure can increase glomerular filtration rate (15). In addition, the role of mannitol as a protective agent in AKI is based on the release of intrarenal vasodilating prostaglandins and natriuretic peptides as well as on its oxygen-free radical scavenger properties (28).

### ICU Indications

One classical renal indication for the use of mannitol was the prevention of AKI because of its potential effect in the removal of obstructing tubular casts, dilution of nephrotoxic substances in the tubular fluid, and reduction in the swelling of tubular elements *via* osmotic extraction of water. Mannitol has been recommended by some authors for the treatment/prevention of rhabdomyolysis-associated AKI, when a urinary output of 300 ml/h or higher is not achieved despite adequate fluid resuscitation (29). Its properties as a myoglobin-related oxygen-free radical scavenger may support its use in this condition. However, current knowledge states that mannitol use is not superior to saline therapy alone (30). Collectively, there is no current evidence that administration of mannitol gives additional benefit beyond adequate hydration in patients at increased risk of AKI, and even for contrast-induced nephropathy, the use of mannitol may be detrimental (31). A recent systematic review and meta-analysis of mannitol as prevention of ischemia–reperfusion injury in kidney transplantation showed a decrease in the incidence of AKI and delayed graft function, an effect possibly beyond its diuretic effect since an increase in urinary output was not observed in the evaluated studies (32). A survey reported in that study disclosed that mannitol is prescribed in about two-thirds of renal transplant units worldwide. The use of mannitol in neurocritically ill patients is also common in the presence or for the prevention of intracranial hypertension; however, the discussion of such use is beyond the scope of this review (33).

In patients with fluid overload, mannitol is contraindicated due to its extracellular expansion effect, which can precipitate pulmonary edema in susceptible patients (15).

We can conclude with current evidence that its renal indication should be focused on kidney transplant surgery, where there is wider evidence.

### Adverse Effects

Even though mannitol does not interfere directly with electrolyte physiologic mechanisms, it can inhibit water transport and consequently decrease the tubular ability to reabsorb Na^+^ and thus lead to hyponatremia. In addition, the increased distal delivery of Na^+^ ions can lead to K^+^ loss. According to the subsequent increase in serum osmolality, mannitol expands the intravascular volume, which can further result in dilutional hyponatremia. If high doses of mannitol are used, hyperkalemia can be observed due to the movement of K^+^ out of the cells (34).

It is important to note that high accumulated doses of mannitol (>200 g per day or accumulated doses of >800 g) have been associated with AKI due to renal vasoconstriction and tubular toxicity, a condition known as osmotic nephrosis. During the time mannitol is being administered, plasma osmolality and the osmolal gap (i.e., the difference between the measured and calculated serum osmolality) should be monitored frequently and therapy should be discontinued if adequate diuresis is not achieved or if the osmolal gap rises above 55 mOsm per kilogram (35, 36).

## Loop Diuretics

Furosemide is the diuretic of choice in more than 90% of the cases of AKI with fluid overload. Numerous studies have demonstrated that use of loop diuretics to achieve greater volume removal is associated with improved outcomes since they can also be useful for reducing the severity of hyperkalaemia and acidosis. Although some studies have suggested that loop diuretics can worsen AKI outcome and increase mortality, these studies are probably flawed with the inclusion of cases of hypovolemic AKI (37). In fact, this suggestion has been rejected by numerous randomized studies (38). As an opposite misconception, theoretical benefits for loop diuretics have been described, such as producing a “resting state” in cells of thick ascending loop of Henle *via* Na/K/2Cl pump inhibition, which could be a therapy for AKI prevention. Unfortunately, there is no evidence of lower mortality or faster kidney function recovery with loop diuretics in patients with AKI (39), although when used in the context of fluid overload, loop diuretics may help in the recovery of AKI through alleviation of renal congestion (40, 41). More data will be necessary to confirm these findings.

### Mechanism of Action

Loop diuretics act on the thick ascending limb of the loop of Henle, blocking the secondary active transport of NaK2Cl (NKCC-2) out of the tubule lumen and producing natriuresis that is accompanied by diuresis, with greater loss of water than sodium, resulting in the production of hypotonic urine. This is accomplished especially by the disruption of the osmotic gradient in renal medulla that reduces the ADH-dependent concentrating ability of the collecting ducts. Loop diuretics arrive at the tubule lumen through active secretion *via* the human organic anion transporter (h OAT) system in the proximal tubule from where it is transported to the luminal NaK2Cl receptor (42).

### Administration and Dosage

There has been controversy about which strategy—either bolus or continuous infusion—is better. The delivery time of loop diuretics to its receptor within the tubular lumen correlates well with diuretic response in both animal and human models (4). Continuous infusion produces less variability in peak plasma furosemide concentration resulting in a consistent plasma drug concentration, with a more predictable diuretic effect. Theoretically, that could reduce diuretic resistance and side effects (dyselectrolytemia and ototoxicity) (43).

RCTs mostly in the context of decompensated heart failure show that there is no difference between continuous infusion and bolus of furosemide for all-cause mortality, length of hospital stay, and electrolyte disturbance, but continuous infusion is superior to bolus administration with regard to diuretic effect (44, 45).

Before starting continuous infusions, if severe oliguric AKI is detected, furosemide stress test (FST) is recommended before continuous infusion as a functional test to predict progressive AKI and in order not to delay RRT initiation (46, 47). The test consists of a one-time dose of 1.0 or 1.5 mg/kg depending on prior furosemide exposure. The cutoff for predicting AKI progression during the first 2 h following FST is a urine volume of less than 200 ml (100 ml/h) with a sensitivity of 87.1% and a specificity 84.1%. The 2-h time is based on the duration of natriuretic effect (theoretically 6 h after oral administration and 2 h after intravenous administration) (46). Since the publication of the initial pilot study in 2013, there have been retrospective and prospective validations of this cutoff (47). Also, this initial bolus or “loading dose” allows continuous loop diuretic infusions to immediately reach peak effective levels that may be critically important in patients with severe congestive heart failure.

### Furosemide Resistance in ICU

The development of diuretic resistance in ICU depends on post-diuretic sodium retention, reduced tubular secretion of the drug, and reduced sodium/chloride sensing.

The increase in Na reabsorption in the distal and collecting ducts offsets the blockade of Na reabsorption in the loop of Henle. This phenomenon is more frequent in chronic loop diuretic therapy because of the hypertrophy and hyperplasia that is developed with time in the epithelial cells of the distal tubules (48). This problem can be handled with the addition of distal duct diuretics (more information in *Section 5*).

Another mechanism of diuretic resistance in the ICU is hypoalbuminemia, which impairs furosemide delivery to the proximal tubule lumen and affects drug efficacy since furosemide is 95% bound to albumin in plasma. Administration of intravenous albumin with furosemide can increase urine output after albumin administration, but its use is controversial. A recent metanalysis including 13 RCTs revealed that furosemide with albumin co-administration increased urine output by 31.45 ml/h in comparison to furosemide treatment alone. The diuretic effect was better in participants with low baseline serum albumin levels (<2.5 g/dl), high prescribed albumin infusion doses (>30 g), and in those with baseline Cr >1.2 mg/dl. The effect was greater within 12 h after administration. Of note, high heterogeneity across studies was detected (49). No studies have shown benefit in patients with normal blood protein levels.

Also, hyponatremia and hypochloremia are known to correlate with diuretic resistance since chloride regulates diuretic sensitive tubular sodium transporter and low sodium could decrease the diuretic effect (50). High-saline solutions have been proposed as a possible therapeutic solution. In patients with acute decompensated heart failure, the simultaneous use of hypertonic saline solution and furosemide is more effective than furosemide alone, as they correct hyponatremia and hypochloremia (50) Otherwise, the optimal dose, duration, and concentration combination of this approach remain undetermined since study protocols are not uniform. In the largest study conducted by Paterna et al. in 2011, the combination of 150 ml of NaCl 1.4%–4.6% (depending on Na concentration) during 30 min with high doses of furosemide was proposed (51).

### Other Loop Diuretics

When compared to furosemide, torasemide and bumetanide have a more favorable pharmacokinetic profile because of their greater bioavailability, but their use is uncommon in the ICU. In that scenario, differences seem less important since oral diuretics are not frequently used (**Table 2**) (20–22, 52).

**Table 2:** Loop diuretics characteristics and dosage.

Characteristic	Furosemide	Bumetanide	Torsemide
Bioavailability (%)	10–100	80–100	80–100
Affected by food	Yes	Yes	No
Half-life (h) In renal dysfunction In hepatic dysfunction In heart failure	1.5–2 2.8 2.5 2.7	1 1.6 2.3 1.3	3–4 4–5 8 6
Onset (min) oral	30–60	30–60	30–60
Onset (min) intravenous	5	2–3	10
Relative potency compared to furosemide	1 (40 mg oral = 20 mg ev)	40 (1 mg)	2 (20 mg oral =20 mg ev)
Dosage	Start 40 mg IV Maximum recommended dose: 20 mg/h if continuous infusion	Start 1 mg IV Maximum recommended dose: 2 mg/h if continuous infusion)	Start 20 mg Maximum recommended dose 20 mg/h if continuous infusion)

Only one randomized clinical trial assessed the effects of torasemide versus furosemide on renal function in cardiac surgery patients recovering from AKI after continuous renal replacement therapy. Both drugs were effective in increasing urine output, but torasemide showed a better dose-dependent diuretic effect (53). As for patients with heart failure, there is evidence to support the use of torasemide over furosemide in non-hospitalized patients. Through additional anti-aldosterone properties, torasemide decreases left-ventricular remodeling, mortality, and hospitalizations compared with furosemide (54). Nevertheless, there are no trials that have included patients in the ICU.

## Distal Convoluted Tubule Diuretics (Thiazides and Thiazide-Like Diuretics)

### Mechanism of Action

These drugs act on the distal convoluted tubule (DCT) by blocking the Na-Cl cotransporter (NCC), increasing the excretion of sodium, chloride, and potassium. In addition, some molecules of these families have a certain inhibitory effect on CA, increasing its natriuretic effect (e.g., chlorthalidone). Secondary to this inhibition, greater amounts of sodium and chloride reach the collecting duct, leading to an increase in Na reabsorption through the Cl-independent apical epithelial sodium channel (ENaC), causing a luminal negativization that stimulates the secretion of K^+^ ions and H^+^. On the other hand, there is a conservation of calcium due to increased reabsorption, such as Na^+^, in the proximal tubule as a compensatory mechanism as well as an increase in calcium reabsorption in the distal tubule due to the increase in the 3 Na^+^/Ca2^+^ counter-transporter of the basolateral membrane (promoted by the decrease in intracellular sodium) and by the opening of cationic voltage-gated channels due to intracellular negative polarization [mechanism mediated by “transient receptor potential channel subfamily V member 5” (TRPV5) (15)]. Pharmacokinetic characteristics and dosage of thiazide and thiazide-like diuretics are described in **Table 3** (20–22).

**Table 3:** Pharmacokinetic characteristics and dosage of thiazide and thiazide-like diuretics.

Drug	Oral bioavailability	Relative potency	Estimated half-life	Estimated time of effect	Dosage
Hydrochlorothiazide	70%	1 (25 mg)	2.5 h	12–18 h	12.5–100 mg qd/bid Max 200 mg
Chlorothiazide	9%–56% (dose-dependent)	0.1 (250 mg)	1.5 h	6–12 h	250–500 mg qd/bid Max 1000 mg
Bendroflumethiazide	100%	10 (2.5 mg)	3–10 h	18 h	2.5–10 mg qd/bid Max 10 mg
Chlortalidone	65%	1 (25 mg)	47 h	24–72 h	12.5–100 mg qd Max-100 mg
Indapamide	93%	20 (1.25 mg)	14 h	24–36 h	2.5–5 mg qd Max 5 mg
Metolazone	65%	10 (2.5 mg)	NA	24 h	2.5–20 mg qd Max 20 mg

Qd, once a day; Bid, twice a day.

### Potential ICU Indications

This diuretic family is the most commonly used when loop diuretic resistance is present in patients with fluid overload. Côté JM et al. reviewed a database of 7,645 ICU patients requiring more than 1 mg/kg/day of furosemide (MIMIC-III database) and found that nearly half of patients who received continuous loop diuretic infusion or intravenous albumin during their ICU stay also received a second class of diuretic, and thiazide plus loop diuretic was the most frequent combination. The net effect was an increase in 24-h urine output similar to that produced with CAI, but use of thiazides was associated with a greater weight loss at 48 h. On the other hand, a substantial increased risk of electrolyte disturbances, mainly hypokalemia, was found (55). The indication of this combination for increasing urine output and negative fluid balance is also recommended by European Heart Failure Guidelines when diuresis remains inadequate with loop diuretics (56).

The presence of ICU-acquired hypernatremia (IAH) is another theoretical potential indication in the ICU setting. IAH appears mainly from disturbances in water and sodium homeostasis, including salt overloading and inadequate water administration. Hypernatremia might help in specific scenarios such as the treatment of cerebral edema by raising serum osmolarity but the degree of hypernatremia that could be achieved without leading to excess mortality is unknown (57). Unfortunately, van IJzendoorn et al. conducted the first placebo-controlled trial that found that 25 mg of hydrochlorothiazide did not significantly affect serum Na or urinary Na in patients with IAH (58).

We can conclude that DCT diuretics should be the first option when furosemide resistance in fluid overloaded patients in the ICU, especially in acute heart failure and also to be considered when hypernatremia is present.

### Adverse Effects

The potential adverse events are related mainly due to electrolyte changes (hyponatremia, hypokalemia, metabolic alkalosis, and hypercalcemia) secondary to its mechanism of action. In particular, attention should be paid to hyponatremia, when thiazides are used in combination with upstream diuretics. As they do not affect the medullary osmotic gradient, thiazide do not impair the ADH-dependent concentrating ability of the collecting ducts, and this leads to higher net sodium loss in comparison with water. Attention should also be paid to allergic reactions as they also have a cross-reactivity with sulfonamides (27) (more information in Section 2).

## Distal Potassium-Sparing Diuretic Agents

### Mechanism of Action

This pharmacological group includes those molecules that directly block ENaC (amiloride and triamterene) and the mineralocorticoid receptor antagonists (spironolactone and eplerenone). They act on the distal cells of the DCT, connecting tubule, and on the cortical portion of the collecting tubule (ASDN) inhibiting Na^+^ reabsorption through ENaC, causing a decrease in K^+^ and H^+^ secretion by depolarizing the negative luminal-transepithelial gradient. Furthermore, amiloride and triamterene reduce the urinary excretion of Ca^2+^ and Mg^2+^. By acting at such a distal level, where Na^+^ reabsorption represents <3%, its diuretic effect is lower compared to those described previously. A peculiarity of spironolactone and eplerenone is that they are the only diuretics that do not need to be secreted into the luminal portion of the tubules to exert their effect (15). Pharmacokinetic characteristics and dosage of potassium-sparing diuretics can be found in **Table 4** (20–22).

**Table 4:** Pharmacokinetic characteristics and dosage of potassium-sparing diuretics.

Drug	Oral bioavailability	Relative potency	Estimated half-life	Estimated time of effect	Dosage
Triamterene	50%	1 (50 mg)	2–5 h	2 h	100–150 mg bid Max 300 mg
Amiloride	15%–25%	10 (5 mg)	21 h	6 h	5–20 mg qd
Spironolactone	65%	NA	1.6 h (15 h active metabolites)	24–48 h	12.5–50 mg qd Max 50 mg
Eplerenone	NA	NA	5 h	NA	25–50 mg qd Max 50 mg

Qd, once a day; Bid, twice a day.

### Potential ICU Indications

In the field of intensive care, these drugs are not used regularly due to their poor diuretic effect and the risk of developing serious complications such as hyperkalemia. Experimental data have shown that administration of spironolactone could prevent renal injury induced by ischemia–reperfusion in rats (59), but Barba-Navarro et al. carried out a randomized placebo-controlled trial in which spironolactone or placebo was administered before and after cardiac surgery and found that the incidence of AKI was higher for the spironolactone group (60).

Furthermore, in the study conducted by Côté et al. from the MIMIC-III database, the combination of these drugs with furosemide was actually associated with a reduction in the 24-h urine output with a reduced risk of hypokalemia and without increasing the incidence of hyperkalemia (55).

We can conclude that these drugs should not be used to treat congestion or to prevent AKI but could play a role when congestion is under control but persistent hypokalemia exists due to other diuretics.

### Adverse Events

The most frequent complication of this diuretic group is the development of hyperkalemia, this being dose-dependent and with a higher risk in patients with CKD or those taking potassium supplements, angiotensin-converting enzyme/angiotensin receptor blockers, anti-inflammatory drugs, beta-blockers, heparin, or ketoconazole. It can also cause metabolic acidosis. Amiloride and triamterene accumulate in renal failure while triamterene accumulates in liver cirrhosis; thus, they should be avoided in such scenarios. A noteworthy effect of triamterene is that it can precipitate in the urinary collecting system and cause obstruction at that level (27).

## Vasopressin Receptor Antagonist

### Mechanism of Action

Vasopressin receptor antagonists act at the most distal level of the nephron, specifically by inhibiting the vasopressin V2 receptors (ADH) of the principal cells of the collecting duct, whose function consists mainly of solute-free water reabsorption, being a fundamental mechanism of the renal ability to concentrate urine, through the activation of a cAMP-mediated pathway whose result is the transport of aquaporins to the luminal membrane with the subsequent reabsorption of free water. For this reason, they are often known as “aquaretics”. There are three receptors in the body for antidiuretic hormone or vasopressin (ADH). These molecules exert their action by competitively inhibiting such receptors: conivaptan on V1a/V2 while tolvaptan, mozavaptan, and lixivaptan act selectively against V2, and have the advantage of being administered orally. The only ones currently approved are conivaptan and tolvaptan (15).

### Potential ICU Indications

Excluding euvolemic hyponatremia due to SIADH, which is not the focus of this review, its main indication is hypervolemic hyponatremia refractory to fluid restriction. There is a lack of evidence in the ICU setting but evidence in hospitalized patients support its use to correct hyponatremia (61).

Loop diuretics may exacerbate sodium and water imbalance and worsen hyponatremia. Fluid restriction, the first step to control hyponatremia, is often difficult to achieve in clinical practice and aquaresis presents an advantage over other diuretics, since removal of water is not accompanied by elimination of sodium and other electrolytes.

In the outpatient setting, SALT 1 and SALT2 randomized placebo-controlled trials examined the effect of tolvaptan on hypervolemic and euvolemic hyponatremia of diverse causes in 448 patients. They were conducted primarily without mandated fluid restriction or a change in the patient’s medication regimen, such as use of diuretics. Tolvaptan was effective in increasing serum sodium concentrations as well as urinary output at day 4 and day 30. The percentage of patients who were under other diuretic treatment was not specified (62).

In a hospitalized non-ICU setting, the EVEREST randomized placebo-controlled trial that included 4,133 patients evaluated the effects of tolvaptan initiated in patients hospitalized with heart failure. Primary end points were all-cause mortality (superiority and noninferiority) and cardiovascular death or hospitalization for heart failure (superiority only). Secondary end points included changes in dyspnea, body weight, and edema. All patients received standard heart failure therapy, including diuretics. Tolvaptan had no effect on long-term mortality or heart failure–related morbidity. It improved hyponatremia, dyspnea, edema, and body weight, and serum sodium with renal function preservation (61).

There are currently no trials that have assessed tolvaptan in the ICU scenario; however, some authors support its use for increasing diuresis without major hydroelectrolytic alterations. Ruiz-Ramos et al. reported the first case series of six ICU patients who had fluid overload and used tolvaptan during the recovery phase of septic shock. Five patients were previously treated with >60 mg of furosemide without achieving negative fluid balance. The authors described that all patients achieved an increase in diuresis after tolvaptan use, without important adverse events (63).

We can conclude that tolvaptan can have a role in the treatment of fluid overload with hyponatremia, as it can improve hyponatremia while urinary output is increased.

### Adverse Effects

The most frequently observed side effects are derived from its mechanism of action, mainly thirst, dry mouth, and polyuria. Episodes of ventricular tachycardia have also been described. It has been described to cause liver damage; thus, it is recommended to avoid it in patients with known liver disease (27).

## New Drugs—Sodium-Glucose Cotransporter-2 Inhibitors (SGLT2i) or Gliflozines

### Mechanism of Action

Gliflozines are a novel class of antidiabetic drugs that have demonstrated great benefits regarding cardiovascular and kidney protection as well as in the treatment of heart failure with or without diabetes (64, 65). They act by inhibiting the sodium-glucose cotransporter-2 (SGLT-2), located in the proximal tubule, and responsible for the reabsorption of 90% of filtered glucose. Because of this, they promote glucosuria and thereby increase urine osmolality, forcing an osmotic effect, increasing by this way diuresis. Moreover, while inhibiting this cotransporter, coupled with sodium, this cation reabsorption is also supressed, leading to natriuresis. It has been proposed that natriuresis would also result from a decrease in Na^+^/H^+^ exchanger (NHE3) and bicarbonate flux in the proximal tubule, mimicking acetazolamide’s mechanism of action (66). As a result, theoretically, by these concomitant mechanisms, SGLT2i increases diuresis and therefore can reduce extracellular fluid volume by natriuretic and osmotic diuretic effect. However, in patients treated with SGLT2, the increase of diuresis is reduced or even disappears after 72 h, probably due to activation of urinary concentration through stimulation of urea production and vasopressin action (67). Therefore, the proven cardiorenal protection of these agents may rely not only on its mere osmotic diuresis but also perhaps through the activation of specific metabolic pathways driven by the energy-wasting glycosuria, which may benefit function and longevity (68).

### ICU Indications

SGLTi is such a novel treatment that its indications are restricted to the treatment of T2DM and, most recently approved, the treatment of heart failure with reduced ejection fraction, both in the ambulatory setting. In patients hospitalized for acute conditions, there is a risk for euglycemic ketoacidosis with these drugs (see below) (69). Therefore, more studies will be necessary to demonstrate organ protection in acute patients. In a recent randomized placebo-controlled trial of patients admitted for COVID-19 infection, the administration of dapagliflozin showed a trend to multiorgan protection, including AKI. Out of the 1,250 included patients, 3.8% in the dapagliflozin group vs. 5.6% in the placebo group had AKI during admission (70).

In the heart failure field, DAPA-HF and EMPEROR-Reduced placebo controlled trial included patients with HYHA II–IV with reduced ejection fraction and proved that the risk of worsening heart failure or death from cardiovascular causes was lower among those who received dapagliflozin and empagliflozin, respectively^64,65.^ In both studies, patients were receiving the standard treatment, which could include diuretics. The drug also proved to be beneficial in the renal field in patients with and without diabetes. The recently published DAPA-CKD study included patients with eGFR 25–75 ml/min and albumin/creatinine ratio 200–5,000 mg/g with or without diabetes, and they found that the risk of CKD progression was significantly lower in those treated with dapagliflozin compared to placebo with a median follow-up of 2.4 years (71).

This combination of heart and renal benefits makes this diuretic a promising tool for hospitalized patients too, where other diuretics are commonly used. A recent randomized placebo-controlled crossover study conducted by Griffin et al. included 20 hospitalized patients with type 2 diabetes and chronic stable heart failure and found that following the administration of bumetanide, a significant synergistic effect on natriuresis was observed in patients receiving empagliflozin both during the day 1 and day 14 visits. They propose that the cardiovascular and nephroprotective effects of this drug are mediated by the natriuretic action of SGLT2i instead of the glucose control (72). On the other hand, a substudy of the EMPA-RESPONSE-AHF placebo-controlled trial and, for the first time, including hospitalized patients did show an increase in plasma osmolality and glycosuria but without affecting FeNa. This study did not specifically analyze patients under loop diuretic treatment, but distribution was equal between groups (73).Considering all of this, we need more studies in hospitalized patients with fluid overload in order to analyze their diuretic power.

### Adverse effects

The most common side effects we are aware of are urinary tract infections and genital mycotic infections. Two important adverse events that have been described are the major incidence reported for Fournier gangrene (necrotizing fasciitis of the perineum) and the development of euglycemic diabetic ketoacidosis (74).

## Epidemiology of Diuretic Use in the ICU

Diuretics are the main tool to treat fluid overload, but there are few guidelines regarding the most appropriate diuretic strategy in critically ill patients. McCoy et al. (75 *)* studied the patterns of diuretic use in ICU in the largest contemporary database. They analyzed 46,037 adult ICU patients from 2001 to 2012 and found that nearly half of the patients took diuretics during admission. The most common diuretic was furosemide in 94.4% of the patients and only 22.1% used combined diuretics at some point. The median furosemide dose was 20 mg and only increased to 40 mg when serum creatinine was >3 mg/dl. Fourteen percent of the furosemide use was in continuous infusion, but interestingly, 30% did not receive a bolus on the day of infusion initiation despite the recommendation of a loading dose to allow continuous infusion to work faster (47). Diuretic combination was more common in cardiac units where loop diuretics + thiazide combination was given to 6.1% of ICU admissions, probably regarding the European Heart Failure Guideline recommendations (56). This study reflects that diuretic combinations are not common in ICU probably because of concern about dyselectrolytemia, despite being a well-known method of overcoming loop diuretic resistance.

Moving to diuretic resistance, Cote used the same database to analyze ICU adults requiring more than 1 mg/kg/day of furosemide in order to describe the efficacy and safety of different pharmacologic strategies to promote negative fluid balance in critically ill patients (55). When it comes to diuretic combination, out of the 6,358 patients included, 1,811 received a second diuretic, with thiazides being the most common (62%) followed by CAI and distal potassium-sparing diuretics. Always compared with loop diuretic bolus alone, in terms of efficacy, the combination of thiazides and CAI was associated with both higher urine output (*p* < 0.001) and weight loss at 48 h (*p* < 0.01). On the other hand, the most effective diuretic strategy in terms of fluid balance and urinary output at 24 h was loop diuretic continuous infusion, but this advantage was not maintained at 48 h, where results tended to be similar to combined diuretics. This finding seems to be in agreement with thiazides’ delayed peak of action compared to loop diuretics. In terms of safety, the risk of severe hyponatremia events was more frequent with thiazide combination (OR: 1.71, 95% CI: 1.08–2.69) and for severe hypokalemia (OR: 2.28, 95% CI: 1.70–3.06), despite adjustment for the dose of potassium supplements. The coadministration of a loop diuretic with a distal potassium-sparing diuretics was associated with a reduced risk of hypokalemia without any change in the risk of hyperkalemia. As for CAIs, they also increased the risk of hypokalemia (OR: 1.81, 95% CI: 1.57–2.10) and reduction of bicarbonate and pH by −0.02. They also observed that continuous infusion was associated with hypokalemia (OR: 1.60, 95% CI: 1.47–1.74) and increase in serum chloride. This study shows that despite the fact that continuous loop diuretic infusion seems to be the more effective in terms of fluid balance, diuretic combination can play a role in maintaining fluid balance after 48 h. Thiazides should be avoided when hyponatremia and hypokalemia are expected during continuous infusion and combination strategies. In this study, the acetazolamide–loop diuretic combination was associated with a similar efficacy compared to the more commonly used thiazide–loop diuretic combination but reduced serum bicarbonate and pH while increasing serum chloride, such that adverse events should be considered before prescription.

**Figure 2:**
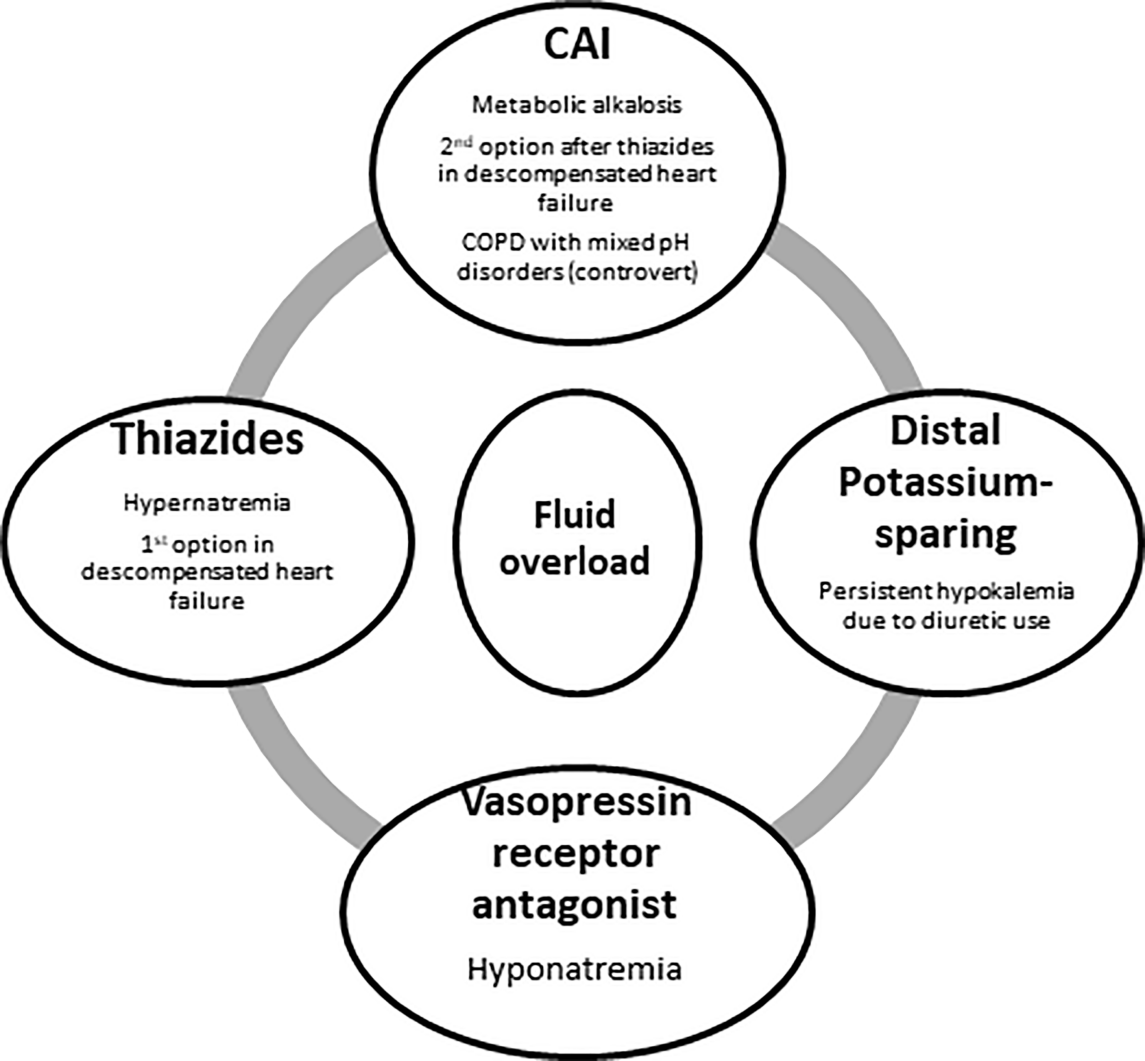
Potential indications for the combination of non-loop diuretics in the case of fluid overload in the ICU.CAI, carbonic anhydrase inhibitors.

Our diuretic combination recommendation can be found in **Figure 2**, and a summary of diuretics use in fluid overload is shown in **Table 5**.

**Table 5:** Summary of diuretics use in fluid overload.

Diuretic	CAI	Loop	DCT	K-sparing	VRA
Indication	Metabolic alkalosis Add-on therapy when loop resistance, 2nd option after thiazides in descompensed heart failure COPD with mixed pH disorders (controversial)	1st option	Hypernatremia 1st add-on therapy option when loop resistence in descompensed heart failure	Persistent hypokalemia due to diuretic use	Hyponatremia
Adverse events	Dyselectrolytemia (specially hypokalemia and hyponatremia) Metabolic acidosis Sulfonamide family reactions	Sulfonamide family reactions (less common) Dyselectrolytemia (hypokalemia, hypocalcemia, hypomagnesemia, hypernatremia) Metabolic alkalosis	Dyselectrolitemia (specially hyponatremia, hypokalemia, hypercalcemia) Metabolic alkalosis Sulfonamide family reactions (less common)	Dyselectrolitemia (specially hyperkalemia) Metabolic acidosis	Liver damage Hypernatremia
Cautions for usage	Monitor ionogram and acid–base status Avoid in advance liver disease	Monitor ionogram and acid–base status Ototoxicity (if high dose and quick infusion)	Monitor ionogram and acid–base status Avoid in advance liver disease	Triamterene: avoid in advanced kidney and liver disease Amiloride: avoid in advanced kidney disease Monitor K^+^, specially if in combination with ACEI/ARB	Monitor sodium levels Avoid in advanced liver disease

CAI, carbonic anhidrase inhibitors; DCT, distal convoluted tubule; VRA, vasopressin receptor antagonists; ACEI, angiotensin-converting enzyme inhibitors; ARB, angiotensin II receptor blockers; COPD, chronic obstructive pulmonary disease.

### Diuretic Use in Special Population

Cirrhotic patients are a special population in which diuretic use to treat congestion remains a challenge in ICU (76, 77). Splanchnic arterial vasodilation due to portal hypertension leads to reduced effective volemia and the subsequent sodium retention due to the activation of sodium-retaining systems, such as the renin–angiotensin–aldosterone system (RAAS) and sympathetic nervous system, which is the mainstay of ascites formation.

Before starting treatment, a diagnostic paracentesis is recommended in all patients with new-onset ascites to rule out spontaneous bacterial peritonitis. Diuretics are needed to counteract renal sodium retention in decompensated cirrhosis with ascites and edema. As secondary hyperaldosteronism is a major pathogenetic mechanism, aldosterone antagonists should be first-line diuretics (starting dose 100 mg/day, increased to 400 mg/day). Furosemide is added in order to increase urinary output (starting dose 40 mg/day, increased to 160 mg/day).

Adverse events related to diuretic therapy are common in cirrhotic patients, requiring drug discontinuation or dose reduction. The most commonly reported adverse events in ICU patients are hepatic encephalopathy, renal impairment, and hyponatremia. In order to avoid these adverse events, the recommended negative fluid balance induced by diuretics should not lead to a body weight loss exceeding 0.5 kg/day in patients without peripheral edema and 1 kg/day in the presence of peripheral edema in order to avoid plasma volume contraction.

Even though the use of albumin is the gold standard after large-volume paracentesis in order to prevent complications associated with the technique, its use in the case of diuretic resistance has not been proven. Two RCTs have explored the use of long-term administration of albumin in order to improve control of ascites and survival, with contradictory findings (78, 79). Once ascites is refractory to diuretics, guidelines recommend to discontinue them. When renal sodium excretion on diuretics exceeds 30 mmol/day, maintenance of diuretic therapy can be considered, if well tolerated.

As for nephrotic syndrome, there are no adult guidelines available on the management of edema and volume overload, which is also uncommonly treated in the ICU. There are two models of edema formation in nephrotic syndrome, the so-called under-filling and the over-filling models, both resulting in sodium and water retention and increased interstitial fluid volume. The under-filling model proposes that the decreased circulating volume due to hypoalbuminemia leads to renal hypoperfusion and activation of the RAAS, and this stimulation causes sodium and water reabsorption. The over-filling model states that changes in the capillary endothelial filtration barrier are responsible for sodium and water rearbsorption (20).

There is no consensus approach to diuretic treatment. It is recommended to use loop diuretic in combination with DCT. Loop diuretics should be started at a low dose and then sequentially increased until optimal weight loss or until maximum dose. If, at this stage, the patient remains symptomatic, a DCT should be added.

Replacing serum albumin with intravenous infusions to improve the efficacy of loop diuretics has been investigated. Hypoalbuminemia reduces the amount of loop diuretic that is delivered to the tubular lumen so albumin infusion may help overcome diuretic resisteance. On the other hand, complications due to albumin infusion (especially hypertension) are not uncommon, and this leads to recommending albumin infusion only in those patients with diuretic resistance and albumin levels <2 g/dl. The recommended proportion is 10 g of albumin for each 40 mg of furosemide.

We can conclude that considering the potential benefits of non-loop diuretics, ICU doctors can take advantage of them by their use in a particular patient profile.

## Author Contributions

VE, JM, and AM-A participated in the conception, design, and drafting of the article. EP and AM-A supervised the work. GP, DC, AC, and AJ provided intellectual content of critical importance to the work and gave their final approval for the version to be published. All authors contributed to the article and approved the submitted version.

## Conflict of Interest

The authors declare that the research was conducted in the absence of any commercial or financial relationships that could be construed as a potential conflict of interest.

## Publisher’s Note

All claims expressed in this article are solely those of the authors and do not necessarily represent those of their affiliated organizations, or those of the publisher, the editors and the reviewers. Any product that may be evaluated in this article, or claim that may be made by its manufacturer, is not guaranteed or endorsed by the publisher.

## References

[1] Uchino S Kellum JA Bellomo R Doig GS Morimatsu H Morgera S. Acute Renal Failure in Critically Ill Patients: A Multinational, Multicenter Study. JAMA (2005) 294(7):813–8. doi: 10.1001/jama.294.7.813 16106006

[2] Ronco C Bellomo R Kellum JA. Acute Kidney Injury. Lancet (2019) 394(10212):1949–64. doi: 10.1016/S0140-6736(19)32563-2 31777389

[3] Cecconi M Hofer C Teboul JL Pettila V Wilkman E Molnar Z. Fluid Challenges in Intensive Care: The FENICE Study: A Global Inception Cohort Study. Intensive Care Med (2015) 41(9):1529–37. doi: 10.1007/s00134-015-3850-x PMC4550653 26162676

[4] Rivers E Nguyen B Havstad S Ressler J Muzzin A Knoblich B. Early Goal-Directed Therapy in the Treatment of Severe Sepsis and Septic Shock. N Engl J Med (2001) 345(19):1368–77. doi: 10.1056/NEJMoa010307 11794169

[5] Dellinger RP Carlet JM Masur H Gerlach H Calandra T Cohen J. Surviving Sepsis Campaign Guidelines for Management of Severe Sepsis and Septic Shock. Crit Care Med (2004) 32(3):858–73. doi: 10.1097/01.CCM.0000117317.18092.E4 15090974

[6] Angus DC Barnato AE Bell D Bellomo R Chong CR Coats TJ. A Systematic Review and Meta-Analysis of Early Goal. Directed Therapy for Septic Shock: The ARISE, ProCESS and ProMISe Investigators. Intensive Care Med (2015) 41(9):1549–60. doi: 10.1007/s00134-015-3822-1 25952825

[7] National Heart, Lung, and Blood Institute Acute Respiratory Distress Syndrome (ARDS) Clinical Trials Network Wiedemann HP Wheeler AP Bernard GR Thompson BT Hayden D deBoisblanc B. Comparison of Two Fluid-Management Strategies in Acute Lung Injury. N Engl J Med (2006) 354(24):2564–75. doi: 10.1056/NEJMoa062200 16714767

[8] Messmer AS Zingg C Müller M Gerber JL Schefold JC Pfortmueller CA. Fluid Overload and Mortality in Adult Critical Care Patients-A Systematic Review and Meta-Analysis of Observational Studies. Crit Care Med (2020) 48(12):1862–70. doi: 10.1097/CCM.0000000000004617 33009098

[9] Prowle JR Kirwan CJ Bellomo R. Fluid Management for the Prevention and Attenuation of Acute Kidney Injury. Nat Rev Nephrol (2014) 10(1):37–47. doi: 10.1038/nrneph.2013.232 24217464

[10] Chen X Wang X Honore PM Spapen HD Liu D. Renal Failure in Critically Ill Patients, Beware of Applying (Central Venous) Pressure on the Kidney. Ann Intensive Care (2018) 8(1):91. doi: 10.1186/s13613-018-0439-x 30238174 PMC6146958

[11] Bhardwaj V Vikneswaran G Rola P Raju S Bhat RS Jayakumar A. Combination of Inferior Vena Cava Diameter, Hepatic Venous Flow, and Portal Vein Pulsatility Index: Venous Excess Ultrasound Score (VEXUS Score) in Predicting Acute Kidney Injury in Patients With Cardiorenal Syndrome: A Prospective Cohort Study. Indian J Crit Care Med (2020) 24(9):783–9. doi: 10.5005/jp-journals-10071-23570 PMC7584837 33132560

[12] Beaubien-Souligny W Rola P Haycock K Bouchard J Lamarche Y Spiegel R. Quantifying Systemic Congestion With Point-Of-Care Ultrasound: Development of the Venous Excess Ultrasound Grading System. Ultrasound J (2020) 12(1):16. doi: 10.1186/s13089-020-00163-w 32270297 PMC7142196

[13] Zhao GJ Xu C Ying JC Lü WB Hong GL Li MF. Association Between Furosemide Administration and Outcomes in Critically Ill Patients With Acute Kidney Injury. Crit Care (2020) 24(1):75. doi: 10.1186/s13054-020-2798-6 32131879 PMC7057586

[14] Grams ME Estrella MM Coresh J Brower RG Liu KD National Heart, Lung, and Blood Institute Acute Respiratory Distress Syndrome Network. Fluid Balance, Diuretic Use, and Mortality in Acute Kidney Injury. Clin J Am Soc Nephrol (2011) 6(5):966–73. doi: 10.2215/CJN.08781010 PMC3087792 21393482

[15] Ellison DH Hoorn EJ Wilcox CS. Chapter 50: Diuretics. In: Taal MW Cherlow GM Marsden PA Skorecki K Yu ASL Brenner BM, editors. Brenner and Rectos’s The Kidney, 9th ed. Philadelphia: Saunders Elsevier (2012). p. 1879–916.

[16] Occhipinti R Boron WF. Role of Carbonic Anhydrases and Inhibitors in Acid-Base Physiology: Insights From Mathematical Modeling. Int J Mol Sci (2019) 20(15):3841. doi: 10.3390/ijms20153841 31390837 PMC6695913

[17] Kassamali R Sica DA. Acetazolamide. Cardiol Rev (2011) 6):276–8. doi: 10.1097/CRD.0b013e31822b4939 21983315

[18] Wongboonsin J Thongprayoon C Bathini T Ungprasert P Aeddula NR Mao MA. Acetazolamide Therapy in Patients With Heart Failure: A Meta-Analysis. J Clin Med (2019) 8(3):349. doi: 10.3390/jcm8030349 30871038 PMC6463174

[19] Moyer JH Ford RV. Laboratory and Clinical Observations on Ethoxzolamide (Cardrase) as a Diuretic Agent. Am J Cardiol (1958) 1(4):497–504. doi: 10.1016/0002-9149(58)90121-8 13520609

[20] Seldin DW Giebisch GH. Diuretic Agents: Clinical Physiology and Pharmacology. San Diego: Academic Press (1997).

[21] Arumugham VB Shahin MH. Therapeutic Uses Of Diuretic Agents. In: StatPearls. Treasure Island (FL: StatPearls Publishing (2022). 32491770

[22] Tighe DA Tran MT Donovan JL Cook JR. Cardiology Drug Guide 2010. Sudbury, Massachusetts: Jones and Bartlett Publishing (2010).

[23] Verbrugge FH Martens P Ameloot K Haemels V Penders J Dupont M. Acetazolamide to Increase Natriuresis in Congestive Heart Failure at High Risk for Diuretic Resistance. Eur J Heart Fail (2019) 21(11):1415–22. doi: 10.1002/ejhf.1478 31074184

[24] Anderson LE Henrich WL. Alkalemia-Associated Morbidity and Mortality in Medical and Surgical Patients. South Med J (1987) 80(6):729–33. doi: 10.1097/00007611-198706000-00016 3589765

[25] Heming N Urien S Faisy C. Acetazolamide: A Second Wind for a Respiratory Stimulant in the Intensive Care Unit? Crit Care (2012) 16(4):318. doi: 10.1186/cc11323 22866939 PMC3580678

[26] Faisy C Meziani F Planquette B Clavel M Gacouin A Bornstain C. Effect of Acetazolamide vs Placebo on Duration of Invasive Mechanical Ventilation Among Patients With Chronic Obstructive Pulmonary Disease: A Randomized Clinical Trial. JAMA (2016) 315(5):480–8. doi: 10.1001/jama.2016.0019 26836730

[27] Hardman JG Limbird LE Goodman Gilman A eds. Drugs Affecting Renal Excretory Function. In: Goodman and Gilman’s. The Pharmacological Basis of Therapeutics. New York: McGraw. Hill.

[28] Holt S Reeder B Wilson M Harvey S Morrow JD Roberts LJ 2nd. Increased Lipid Peroxidation in Patients With Rhabdomyolysis. Lancet (1999) 353:1241. doi: 10.1016/S0140-6736(98)05768-7 10217088

[29] Scharman EJ Troutman WG. Prevention of Kidney Injury Following Rhabdomyolysis: A Systematic Review. Ann Pharmacother (2013) 47(1):90–105. doi: 10.1345/aph.1R215 23324509

[30] Somagutta MR Pagad S Sridharan S Nanthakumaran S Arnold AA May V. Role of Bicarbonates and Mannitol in Rhabdomyolysis: A Comprehensive Review. Cureus (2020) 12(8):e9742. doi: 10.7759/cureus.9742 32944457 PMC7490772

[31] Yang B Xu J Xu F Zou Z Ye C Mei C. Intravascular Administration of Mannitol for Acute Kidney Injury Prevention: A Systematic Review and Meta-Analysis. PLos One (2014) 9(1):e85029. doi: 10.1371/journal.pone.0085029 24454783 PMC3891750

[32] Laar SCV Schouten GN IJzermans JNM Minnee RC. Effect of Mannitol on Kidney Function After Kidney Transplantation: A Systematic Review and Meta-Analysis. Transplant Proc (2021) 53(7):2122–32. doi: 10.1016/j.transproceed.2021.07.001 34412911

[33] Cook AM Morgan Jones G Hawryluk GWJ Mailloux P McLaughlin D Papangelou A. Guidelines for the Acute Treatment of Cerebral Edema in Neurocritical Care Patients. Neurocrit Care (2020) 32(3):647–66. doi: 10.1007/s12028-020-00959-7 PMC7272487 32227294

[34] Manninen PH Lam AM Gelb AW Brown SC. The Effect of High-Dose Mannitol on Serum and Urine Electrolytes and Osmolality in Neurosurgical Patients. Can J Anaesth (1987) 34:442. doi: 10.1007/BF03014345 3117392

[35] Better OS Rubinstein I Winaver JM Knochel JP. Mannitol Therapy Revisited (1940-1997). Kidney Int (1997) 52:886–94. doi: 10.1038/ki.1997.409 9328926

[36] Visweswaran P Massin EK Dubose TD Jr. Mannitol-Induced Acute Renal Failure. J Am Soc Nephrol (1997) 8:1028–33. doi: 10.1681/ASN.V861028 9189872

[37] Levi TM Rocha MS Almeida DN Martins RT Silva MG Santana NC. Furosemide is Associated With Acute Kidney Injury in Critically Ill Patients. Braz J Med Biol Res (2012) 45:827–33. doi: 10.1590/S0100-879X2012007500093 PMC3854324 22641414

[38] Krzych ŁJ Czempik PF. Impact of Furosemide on Mortality and the Requirement for Renal Replacement Therapy in Acute Kidney Injury: A Systematic Review and Meta-Analysis of Randomised Trials. Ann Intensive Care (2019) 9(1):85. doi: 10.1186/s13613-019-0557-0 31342205 PMC6656832

[39] Cantarovich F Rangoonwala B Lorenz H Verho M Esnault VLM. High-Dose Furosemide for Established ARF: A Prospective, Randomized, Double-Blind, Placebo-Controlled, Multicenter Trial. Am J Kidney Dis (2004) 44(3):402–9. doi: 10.1016/S0272-6386(04)00810-8 15332212

[40] Ho KM Power BM. Benefts and Risks of Furosemide in Acute Kidney Injury. Anaesthesia (2010) 65:283–93. doi: 10.1111/j.1365-2044.2009.06228.x 20085566

[41] Joannidis M Druml W Forni LG Groeneveld ABJ Honore PM Hoste E. Prevention of Acute Kidney Injury and Protection of Renal Function in the Intensive Care Unit: Update 2017: Expert Opinion of the Working Group on Prevention, AKI Section, European Society of Intensive Care Medicine. Intensive Care Med (2017) 43(6):730–49. doi: 10.1007/s00134-017-4832-y PMC5487598 28577069

[42] Feig PU. Cellular Mechanism of Action of Loop Diuretics: Implications for Drug Effectiveness and Adverse Effects. Am J Cardiol (1986) 57(2):14A–9A. doi: 10.1016/0002-9149(86)91001-5 3511652

[43] Martin SJ Danziger LH. Continuous Infusion of Loop Diuretics in the Critically Ill: A Review of the Literature. Crit Care Med (1994) 22(8):1323–9. doi: 10.1097/00003246-199408000-00017 8045153

[44] Ng KT Yap JLL. Continuous Infusion vs. Intermittent Bolus Injection of Furosemide in Acute Decompensated Heart Failure: Systematic Review and Meta-Analysis of Randomised Controlled Trials. Anaesthesia (2018) 73(2):238–47. doi: 10.1111/anae.14038 28940440

[45] Ostermann M Alvarez G Sharpe MD Martin CM. Frusemide Administration in Critically Ill Patients by Continuous Compared to Bolus Therapy. Nephron Clin Pract (2007) 107(2):c70–6. doi: 10.1159/000108641 17890871

[46] Chawla LS Davison DL Brasha. Mitchell E Koyner JL Arthur JM Shaw AD. Development and Standardization of a Furosemide Stress Test to Predict the Severity of Acute Kidney Injury. Crit Care (2013) 17(5):R207. doi: 10.1186/cc13015 24053972 PMC4057505

[47] McMahon BA Chawla LS. The Furosemide Stress Test: Current Use and Future Potential. Ren Fail (2021) 43(1):830–9. doi: 10.1080/0886022X.2021.1906701 PMC8118439 33971784

[48] Asare K. Management of Loop Diuretic Resistance in the Intensive Care Unit. Am J Health Syst Pharm (2009) 66(18):1635–40. doi: 10.2146/ajhp090068 19729568

[49] Lee TH Kuo G Chang CH Huang YT Yen CL Lee CC. Diuretic Effect of Co-Administration of Furosemide and Albumin in Comparison to Furosemide Therapy Alone: An Updated Systematic Review and Meta-Analysis. PLos One (2021) 16(12):e0260312. doi: 10.1371/journal.pone.0260312 34851962 PMC8635380

[50] Liu C Peng Z Gao X Gajic O Dong Y Prokop LJ. Simultaneous Use of Hypertonic Saline and IV Furosemide for Fluid Overload: A Systematic Review and Meta-Analysis. Crit Care Med (2021) 49(11):e1163–75. doi: 10.1097/CCM.0000000000005174 34166286

[51] Paterna S Fasullo S Cannizzaro S Cannizzaro S Basile I Vitrano G. Short-Term Effects of Hypertonic Saline Solution in Acute Heart Failure and Long-Term Effects of a Moderate Sodium Restriction in Patients With Compensated Heart Failure With New York Heart Association Class III (Class C) (SMAC-HF Study). Am J Med Sci (2011) 342:27–37. doi: 10.1097/MAJ.0b013e31820f10ad 21701268

[52] Brandi T Kristina K Nicholas C. Continuous Infusions of High Dose Bumetanide to Induce Diuresis in Volume Overloaded Critically Ill Cardiac and Cardiothoracic Surgery Patients. Crit Care Med (2012) 40(12):1–328. doi: 10.1097/01.ccm.0000425149.79348.49 23213646

[53] Vargas Hein O Staegemann M Wagner D von Heymann C Martin M Morgera S. Torsemide Versus Furosemide After Continuous Renal Replacement Therapy Due to Acute Renal Failure in Cardiac Surgery Patients. Ren Fail (2005) 27(4):385–92. doi: 10.1081/JDI-200065298 16060124

[54] Wargo KA Banta WM. A Comprehensive Review of the Loop Diuretics: Should Furosemide be First Line? Ann Pharmacother (2009) 43(11):1836–47. doi: 10.1345/aph.1M177 19843838

[55] Côté JM Bouchard J Murray PT Beaubien. Souligny W. Diuretic Strategies in Patients With Resistance to Loop-Diuretics in the Intensive Care Unit: A Retrospective Study From the MIMIC-III Database. J Crit Care (2021) 65:282–91. doi: 10.1016/j.jcrc.2021.06.009 34298494

[56] Bissell BD Laine ME Thompson Bastin ML Flannery AH Kelly A Riser J. Impact of Protocolized Diuresis for De-Resuscitation in the Intensive Care Unit. Crit Care (2020) 24(1):70. doi: 10.1186/s13054-020-2795-9 32111247 PMC7048112

[57] Aiyagari V Deibert E Diringer MN. Hypernatremia in the Neurologic Intensive Care Unit: How High is Too High? J Crit Care (2006) 21(2):163–72. doi: 10.1016/j.jcrc.2005.10.002 16769461

[58] van IJzendoorn MM Buter H Kingma WP Koopmans M Navis G Boerma EC. Hydrochlorothiazide in Intensive Care Unit-Acquired Hypernatremia: A Randomized Controlled Trial. J Crit Care (2017) 38:225–30. doi: 10.1016/j.jcrc.2016.11.035 27984823

[59] Pretorius M Murray KT Yu C Byrne JG Billings FT Petracek MR. Angiotensin-Converting Enzyme Inhibition or Mineralocorticoid Receptor Blockade do Not Affect Prevalence of Atrial Fibrillation in Patients Undergoing Cardiac Surgery. Crit Care Med (2012) 40(10):2805–12. doi: 10.1097/CCM.0b013e31825b8be2 PMC3588582 22824930

[60] Barba-Navarro R Tapia-Silva M Garza-Garcia C López-Giacoman S Melgoza-Toral I Vázquez-Rangel A. The Effect of Spironolactone on Acute Kidney Injury After Cardiac Surgery: A Randomized, Placebo-Controlled Trial. Am J Kidney Dis (2017) 69(2):192–9. doi: 10.1053/j.ajkd.2016.06.013 27522513

[61] Konstam MA Gheorghiade M Burnett JC Jr Grinfeld L Maggioni AP Swedberg K. Effects of Oral Tolvaptan in Patients Hospitalized for Worsening Heart Failure: The EVEREST Outcome Trial. JAMA (2007) 297(12):1319–31. doi: 10.1001/jama.297.12.1319 17384437

[62] Schrier RW Gross P Gheorghiade M Berl T Verbalis JG Czerwiec FS. Tolvaptan, a Selective Oral Vasopressin V2-Receptor Antagonist, for Hyponatremia. N Engl J Med (2006) 355(20):2099–112. doi: 10.1056/NEJMoa065181 17105757

[63] Ruiz. Ramos J Gordon M Cortes MA Broch MJ Ramirez P. Initial Experience of Use of Tolvaptan in Critically Ill Patients With Fluid Overload. J Clin Pharm Ther (2015) 40(3):339–41. doi: 10.1111/jcpt.12255 25753481

[64] McMurray JJV Solomon SD Inzucchi SE Køber L Kosiborod MN Martinez FA. Dapagliflozin in Patients With Heart Failure and Reduced Ejection Fraction. N Engl J Med (2019) 381(21):1995–2008. doi: 10.1056/NEJMoa1911303 31535829

[65] Anker SD Butler J Filippatos G Khan MS Marx N Lam CSP. Effect of Empagliflozin on Cardiovascular and Renal Outcomes in Patients With Heart Failure by Baseline Diabetes Status: Results From the EMPEROR-Reduced Trial. Circulation (2021) 143(4):337–49. doi: 10.1161/CIRCULATIONAHA.120.051824 PMC7834911 33175585

[66] Borges. Júnior FA Silva Dos Santos D Benetti A Polidoro JZ Wisnivesky ACT Crajoinas RO. Empagliflozin Inhibits Proximal Tubule NHE3 Activity, Preserves GFR, and Restores Euvolemia in Nondiabetic Rats With Induced Heart Failure. J Am Soc Nephrol (2021) 32(7):1616–29. doi: 10.1681/ASN.2020071029 PMC8425656 33846238

[67] Eickhoff MK Dekkers CCJ Kramers BJ Laverman GD Frimodt. Møller M Jørgensen NR. Effects of Dapagliflozin on Volume Status When Added to Renin-Angiotensin System Inhibitors. J Clin Med (2019) 8(6):779. doi: 10.3390/jcm8060779 31159350 PMC6616433

[68] Marton A Kaneko T Kovalik JP Yasui A Nishiyama A Titze J. Organ Protection by SGLT2 Inhibitors: Role of Metabolic Energy and Water Conservation. Nat Rev Nephrol (2021) 17:65–77. doi: 10.1038/s41581-020-00350-x 33005037

[69] Lau A Bruce S Wang E Ree R Rondi K Chau A. Perioperative Implications of Sodium-Glucose Cotransporter-2 Inhibitors: A Case Series of Euglycemic Diabetic Ketoacidosis in Three Patients After Cardiac Surgery. Can J Anaesth (2018) 65(2):188–93. doi: 10.1007/s12630-017-1018-6 29168157

[70] Kosiborod MN Esterline R Furtado RHM Oscarsson J Gasparyan SB Koch GG. Dapagliflozin in Patients With Cardiometabolic Risk Factors Hospitalised With COVID-19 (DARE-19): A Randomised, Double-Blind, Placebo-Controlled, Phase 3 Trial. Lancet Diabetes Endocrinol (2021) 9(9):586–94. doi: 10.1016/S2213-8587(21)00180-7 PMC8294807 34302745

[71] Heerspink HJL Stefánsson BV Correa. Rotter R Chertow GM Greene T Hou FF. Dapagliflozin in Patients With Chronic Kidney Disease. N Engl J Med (2020) 383(15):1436–46. doi: 10.1056/NEJMoa2024816 32970396

[72] Griffin M Rao VS Ivey. Miranda J Fleming J Mahoney D Maulion C. Empagliflozin in Heart Failure: Diuretic and Cardiorenal Effects. Circulation (2020) 142(11):1028–39. doi: 10.1161/CIRCULATIONAHA.120.045691 PMC7521417 32410463

[73] Boorsma EM Beusekamp JC Ter Maaten JM Figarska SM Danser AHJ van Veldhuisen DJ. Effects of Empagliflozin on Renal Sodium and Glucose Handling in Patients With Acute Heart Failure. Eur J Heart Fail (2021) 23(1):68–78. doi: 10.1002/ejhf.2066 33251643 PMC8048437

[74] Singh M Kumar A. Risks Associated With SGLT2 Inhibitors: An Overview. Curr Drug Saf (2018) 13(2):84–91. doi: 10.2174/1574886313666180226103408 29485006

[75] McCoy IE Chertow GM Chang TI. Patterns of Diuretic Use in the Intensive Care Unit. PLos One (2019) 14(5):e0217911. doi: 10.1371/journal.pone.0217911 31150512 PMC6544280

[76] Aithal GP Palaniyappan N China L Härmälä S Macken L Ryan JM. Guidelines on the Management of Ascites in Cirrhosis. Gut (2021) 70(1):9–29. doi: 10.1136/gutjnl-2020-321790 33067334 PMC7788190

[77] European Association for the Study of the Liver. Electronic address: easloffice@easloffice.eu European Association for the Study of the Liver. EASL Clinical Practice Guidelines for the Management of Patients With Decompensated Cirrhosis. J Hepatol (2018) 69:406–60. doi: 10.1016/j.jhep.2018.03.024 29653741

[78] Caraceni P Riggio O Angeli P Alessandria C Neri S Foschi FG. Long-Term Albumin Administration in Decompensated Cirrhosis: An Open-Label Randomized Trial. Lancet (2018) 392(10145):386. doi: 10.1016/S0140-6736(18)30840-7 29861076

[79] Sola E Sola C Simon. Talero M Martin. Llahi M Castellote J GarciaMartinez R. Midodrine and Albumin for Prevention of Complications of Cirrhosis in Patients in the Waiting List for Liver Transplantation. A Randomized, Multicenter, Double. Blind, Placebo-Controlled Trial. J Hepatol (2017) 66:S11. doi: 10.1016/j.jhep.2018.08.006 30138685

